# 

**Published:** 1901-09-28

**Authors:** 


### NOTICE TO CORRESPONDENTS.

All MSS., Letters, Books for Review, and other matters intended for the Editor should be addressed The Editor, "The Hospital," The Hospital Building, 28 & 29 Southampton Street, Strand, W.C.

The Editor cannot undertake to return rejected MS., even when accompanied by stamped directed envelope.

**Notice to Advertisers and Subscribers.**

All Advertisements, Orders for Copies of The Hospital, and Business Communications should be addressed to **THE MANAGER (Not to the Editor),** The Hospital, 28 & 29 Southampton Street, Strand, London, W.C.

The Cost of Subscription, post free, is as follows:—Per week, 2½d.; per quarter, 2s. 8d.; per half-year, 5s. 3d.; per annum, 10s. 6d. Foreign:—Per annum, 15s. 2d., payable, in all cases, in advance.

**NOTICE.—Vol. XXX. of "The Hospital" (April 1901, to September 1901), in handsome cloth binding will shortly be on sale at the Office of this Journal, price, 7s. 6d. Cases for binding the Numbers contained in this Volume 1s. 6d. Index to Vol. XXX., 2½d., post free.**

**The Monthly Part for October will be ready on October 1st. Price 6d., by post 8d.**

### SCRAPS AND GLEANINGS.

The total net amount realised by the Cromer Cottage Hospital Extension Fund Bazaar was £507 odd.

This year's annual street collection in York on behalf of the York Hospital and Dispensary resulted in £254 being raised, as against £280 last year.

The new nursing home of the Salisbury Infirmary is to be opened some time in October. The estimated cost of this home and its furniture is £7,885, of which sum £1,575 is still needed.

The result of the street parade in Dumfries, in aid of the Dumfries and Galloway Hospital, which was held on a recent Friday, proved a monetary success, upwards of £170 being collected.

The estimated cost of the proposed infectious diseases hospital to be erected by the Bo'ness Town Council at Bo'ness Acres is £6,000. The plans provide for four blocks of buildings containing 24 beds.

The Lee Memorial Committee have offered, from funds at their disposal, to provide the Salisbury Infirmary with two larger consulting rooms for the out-patient department, which offer has been accepted by the Committee of the Infirmary.

The current annual report of the Committee of Management of the Portsmouth Eye and Ear Infirmary shows that during the year 2,556 new cases were attended as out-patients. Last year's ordinary income amounted to only £548, while the expenditure was nearly £634.

During the year ended June 30, 1900, the number of sea casualties to vessels belonging to the United Kingdom was 5,463, being 1,504 less than during the preceding year. The number of total losses was 420 vessels, being 20 higher than the number for last year.

A fête in aid of the Willesden Cottage Hospital was held at Wembly Park recently, at which there was an exhibition of new and improved hansom cabs. The cabmen with their vehicles arrived on the scene in large numbers and took part in a series of races and trials of skill in driving.

The past year's working of the Salisbury Infirmary shows a deficit of £721, which, added to the adverse balance brought forward from the previous year, made a total indebtedness of £2,095. With the view of wiping off this indebtedness, an earnest personal canvass is to be made in the district.

The home for women inebriates which has been established by the Church of England Temperance Society near King's Lynn has just been opened by the Suffragan Bishop of Ipswich. It provides accommodation for 33 persons, for whom a space of 15 acres is provided for exercise and recreation.

The Irish Cattle Traders' and Stockowners' Association have addressed a letter to the President of the Local Government Board strongly urging that a representative for Ireland should be placed on the Tuberculosis Commission, in view of the fact that live stock is the staple product of the country, and that any legislation affecting the animal industry will have a serious effect upon the future of Ireland.

### The Student of Medicine.

### APPOINTMENTS.

**H. A. Ahrens**, M.R.C.S.Eng., L.R.C.P.Lond., appointed Certifying Factory Surgeon for the Basingstoke District of Hampshire. **Peter Aitken**, L.R.C.P., L.R.C.S.Edin., appointed Certifying Factory Surgeon for the Bo'ness District of Linlithgow. **T. Compton Alwyne**, M.R.C.S., L.R.C.P., appointed Second Assistant Medical Officer, St. Mary Islington Infirmary, Highgate Hill, N. **B. E. G. Bailey**, M.R.C.S., L.R.C.P.Lond., appointed Second Assistant Medical Officer to the Shoreditch Infirmary. **L. W. Bickle**, M.R.C.S., appointed Honorary Surgeon to the Adelaide Hospital, South Australia. **H. J. Blakesley**, F.R.C.S.Eng., appointed Honorary Surgeon to the Leicester Infirmary. **David Crichton**, L.R.C.P., L.R.C.S.Edin., appointed Certifying Factory Surgeon for the Newtyle District of Forfarshire. **A. M. Easterbrook**, M.B., C.M.Edin., appointed Certifying Factory Surgeon for the Gorebridge District of Midlothian. **John Findlay**, M.B., M.Ch.Aberd., appointed Second Assistant Medical Officer to the Dorset County Asylum. **Laurence O. Fuller**, M.R.C.S.Eng., L.R.C.P.Lond., appointed Fourth Assistant Medical Officer at the Darenth Asylum. **A. E. Gibbes**, M.B., appointed Government Medical Officer and Vaccinator at Greta, New South Wales. **Miss E. C. Greenham**, M.B., appointed Resident Surgeon to the Lady Bowen Hospital, Brisbane. **E. H. Hicks**, M.R.C.S., L.S.A., appointed Medical Officer to Wymeswold District of Loughborough Union. **G. W. S. Marr**, M.B., appointed Assistant Medical Superintendent of the Hospital for the Insane at Toowoomba, Queensland. **F. J. Myles**, M.D., appointed Medical Referee under the Workmen's Compensation Act, and Certifying Factory Surgeon for the County of Longford. **N. H. Pike**, M.B., B.S.Lond., appointed District Medical Officer of the Dewsbury Union. **George Read**, L.R.C.S.I., appointed Government Medical Officer and Vaccinator at Picton, New South Wales. **A. Rodger**, M.B., C.M.Edin., appointed Certifying Factory Surgeon for the Yetholm District of the County of Roxburgh. **D. H. Young**, M.B., appointed Officer of Health for Shire of Towong, Victoria.

